# Hyperfiltration in Obesity: The Vicious Link Between Visceral Fat, Proteinuria, and Sodium Excretion

**DOI:** 10.3390/nu18142233

**Published:** 2026-07-09

**Authors:** Mariana Di Lorenzo, Maria Amicone, Andrea Memoli, Nunzia Cacciapuoti, Mariastella Di Lauro, Eleonora Riccio, Antonio Pisani, Bruna Guida, Maria Serena Lonardo

**Affiliations:** 1 Physiology Nutrition Unit, Department of Clinical Medicine and Surgery, University of Naples Federico II, 80131 Naples, Italy; marianadilorenzo.mdl@gmail.com (M.D.L.); nunzia.cacciapuoti.92@gmail.com (N.C.); mariastelladilauro91@gmail.com (M.D.L.); bguida@unina.it (B.G.); 2 Department of Public Health, University of Naples Federico II, 80131 Naples, Italy; ma.amicone.90@gmail.com (M.A.); eleonora.riccio83@gmail.com (E.R.); antonio.pisani13@gmail.com (A.P.); 3 Nephrology Unit, Pellegrini Hospital, 80134 Naples, Italy; andrea_memoli@hotmail.it

**Keywords:** visceral adiposity, chronic kidney disease, obesity, proteinuria

## Abstract

**Background/Objectives:** The global rise in obesity represents a major public health issue, extending its detrimental impact beyond metabolic complications to encompass renal dysfunction. A key element in this relationship lies in the interaction between visceral obesity and glomerular hyperfiltration—an early, often silent indicator of kidney injury that may precede the onset of chronic kidney disease (CKD). **Methods**: In this monocentric, retrospective study, we evaluated 43 adults with obesity attending the Outpatient Clinic for Diet Therapy in Transplantation, Renal Failure and Chronic Pathology at the University of Naples Federico II between March 2022 and March 2024. Clinical, anthropometric, biochemical, and 24 h urinary parameters were recorded, along with several validated visceral adiposity indices. Glomerular hyperfiltration was assessed through measured creatinine clearance (mClCr) tertiles, while associations with adiposity indices, proteinuria, and urinary sodium excretion were explored. **Results**: Higher creatinine clearance values were significantly associated with increased levels of adiposity indices—particularly Lipid Accumulation Product (LAP), New Visceral Adiposity Index (NVAI), and Metabolic Score for Visceral Fat (METS-VF)—as well as greater proteinuria, urinary sodium excretion, and daily salt intake. Subjects with proteinuria ≥ 150 mg/day exhibited higher NVAI values and elevated sodium and potassium urinary excretion compared with those without proteinuria. Significant correlations emerged between LAP and mClCr, sodium excretion, and proteinuria, even after adjustment for age and BMI. **Conclusions**: Higher visceral adiposity indices were associated with increased creatinine clearance, proteinuria, and urinary sodium excretion in adults with obesity. These findings suggest that visceral adiposity may represent a useful marker of early obesity-related renal alterations. Prospective studies are warranted to determine the causal relationships and mechanisms underlying these associations.

## 1. Introduction

Obesity and chronic kidney disease (CKD) are escalating global health challenges with significant clinical and socioeconomic implications. Since 1975, the prevalence of obesity has nearly tripled, with more than 650 million adults now classified as obese [1]. In parallel, CKD affects approximately 10% of the global population and is projected to become the fifth leading cause of death by 2040 [2].

The complex relationship between obesity and CKD has garnered increasing attention, as obesity contributes to kidney disease not only through traditional risk factors such as hypertension and diabetes but also via direct hemodynamic and structural renal effects [3].

Obesity is now recognized as an independent causal factor for CKD. Among obesity-related renal alterations, glomerular hyperfiltration is an early, potentially reversible hemodynamic adaptation that may, over time, predispose to glomerular injury [4]. Recent research highlights that visceral adiposity, rather than generalized obesity, may drive renal dysfunction more strongly [5]. Novel indices estimating visceral fat—such as the Visceral Adiposity Index (VAI), the New Visceral Adiposity Index (NVAI), and the Metabolic Score for Visceral Fat (METS-VF)—provide a more pathophysiologically relevant assessment of obesity-related risk compared to the body mass index (BMI), which fails to capture fat distribution [6].

Given this background, the present study aimed to evaluate the association between renal hyperfiltration, proteinuria, and visceral adiposity indices in adults with obesity.

## 2. Materials and Methods

### 2.1. Study Design and Data Collection

In this monocentric and retrospective study, a total of 43 adults with obesity (16 males; age range: 18–60 years) attending the Outpatient Clinic for Diet Therapy in Transplantation, Renal Failure and Chronic Pathology between March 2022 and March 2024 were consecutively enrolled. All participants provided written informed consent. Inclusion criteria were adult age (18–60 years) and body mass index (BMI) ≥ 30 kg/m^2^. Exclusion criteria included the following: bedridden status, pregnancy or lactation, diabetes mellitus, known kidney or systemic autoimmune diseases, severe infection, anemia, endocrine disorders, cancer or neurological disorders, heart failure, use of ACE inhibitors, angiotensin receptor blockers (ARBs), thiazide or loop diuretics, or SGLT2 inhibitors, and engagement in competitive physical activity.

Demographic, anthropometric, biochemical, and dietary data were collected at baseline by trained clinicians and dietitians.

### 2.2. Anthropometric Measurements and Body Composition Analysis

Anthropometric measurements were obtained according to standardized NIH protocols. Weight and height were measured using a calibrated scale and stadiometer (Seca 711; Seca, Hamburg, Germany). BMI was calculated as weight (kg) divided by height squared (m^2^). Waist circumference (WC) was measured midway between the lower rib margin and the iliac crest using a non-elastic tape.

Body composition was evaluated through bioelectrical impedance analysis (BIA) (BIA 101 RJL, Akern Bioresearch, Florence, Italy) using an 800 μA, 50 kHz current. Measurements were performed under standardized conditions: fasting for at least 6–12 h, abstaining from caffeine, alcohol, and strenuous activity for 24 h prior, and with the subjects lying supine for at least 10 min before electrode placement [7]. Fat Mass (FM), Fat-Free Mass (FFM), and Phase Angle (PA) were recorded.

### 2.3. Clinical and Biochemical Parameters

Blood pressure was measured following the 2021 ESC Guidelines [8]. The mean arterial pressure (MAP) was calculated as one-third Systolic plus two-thirds Diastolic Blood Pressure.

Venous fasting blood samples were collected to determine fasting glucose, glycated hemoglobin (HbA1c), insulin, HOMA-IR, total cholesterol (Tot-C), HDL-C, LDL-C, Triglycerides (TGs), blood urea nitrogen, creatinine, and uric acid using standardized laboratory procedures.

Each subject provided a 24 h urine collection for quantification of albuminuria, proteinuria, sodium, potassium, and creatinine clearance (mClCr). Proteinuria ≥ 150 mg/day represents a clinically meaningful increase above normal urinary protein excretion and warrants monitoring for progressive renal disease [9]. Daily salt intake (g/day) was estimated from 24 h urinary sodium excretion using the standard conversion formula [urinary sodium (mmol/day) × 0.058], according to the protocol reported by Donfrancesco et al. [10].

### 2.4. Adiposity Indices

To assess visceral adiposity beyond BMI, several validated indices were computed, including waist-to-height ratio (WHtR); Visceral Adiposity Index (VAI); New Visceral Adiposity Index (NVAI); Lipid Accumulation Product (LAP); Triglyceride–Glucose Index (TyG); Body Roundness Index (BRI); and Metabolic Score for Visceral Fat (METS-VF).

Detailed formulas for all adiposity indices are provided in Supplementary Table S1 [11,12,13,14,15,16].

### 2.5. Dietary Intake

Dietary intake was assessed using a validated Food Frequency Questionnaire (FFQ) administered by a trained nutritionist during face-to-face interviews. A photographic food atlas containing approximately 1000 standardized portion images was used to improve portion size accuracy [17]. Average daily energy and nutrient intake were calculated using the CREA food composition tables [18].

### 2.6. Statistical Analysis

Statistical analysis was performed using IBM Corp Released 2021, IBM SPSS Statistics for Windows, Version 28.0. Armonk, NY, USA: IBM Corp. For categorical variables, absolute numbers and frequencies (%) are shown.

The Kolmogorov–Smirnoff test was used to check normality. The Levene test was used to evaluate the equality of variance. Normally distributed variables were given as the mean ± standard deviation (SD), and independent-sample *t*-test and analysis of variance (ANOVA) were performed.

For the analysis, patients were stratified into mClCr tertiles, and the highest tertile (mClCr ≥ 159.6 mL/min/1.73 m^2^) was used to define the glomerular hyperfiltration group.

Non-normally distributed variables were expressed as the median and interquartile range (IQR), and non-parametric test of Mann–Whitney and the non-parametric test of multiple comparison of Kruskal–Wallis were carried out.

Partial correlation analysis (adjusted for BMI and age) was used to evaluate the association between adiposity indices and either 24 h urinary sodium excretion (mmol/24 h) and daily salt intake. A formal sample size calculation was not performed, and no correction for multiple testing was applied due to the exploratory nature of the study. The statistical significance was set at *p* < 0.05.

## 3. Results

### 3.1. Study Population Features

A total of 43 individuals with obesity (16 males; 37.2%) were included in the analysis. The mean age was 37 ± 10.7 years, and the mean BMI was 46.3 ± 8.1 kg/m^2^. Baseline demographic, anthropometric, body composition, and clinical and visceral adiposity characteristics of the study population are summarized in Table 1.

All participants were free from overt metabolic or renal disease and were not under pharmacological treatment affecting urinary protein excretion or renal hemodynamics.

### 3.2. Creatinine Clearance Tertiles and Adiposity Indices

When participants were stratified into tertiles of creatinine clearance (mClCr) [Tertile 1 (T1): <121.4 mL/min; T2: 121.4 to <159.6 mL/min; T3: ≥159.6 mL/min], no significant differences were observed across groups regarding age, sex distribution, BMI, body composition parameters, or daily caloric intake (Table 2).

However, increasing mClCr values were accompanied by significantly higher levels of proteinuria as well as elevations in selected visceral adiposity indices. In particular, LAP (*p* = 0.028, η^2^ = 0.167, 95% CI: 41.5–82.3), NVAI (*p* = 0.006, η^2^ = 0.216), and METS-VF (*p* = 0.007, η^2^ = 0.223, 95% CI: 6.8–7.3) values progressively rose across mClCr tertiles. In contrast, VAI, TyG, and BRI did not differ significantly among groups (Table 2).

Furthermore, biochemical analyses revealed that glycated hemoglobin (HbA1c; *p* = 0.004, η^2^ = 0.259, 95% CI: 4.9–5.3), total cholesterol (Tot-C) (*p* = 0.049, η^2^ = 0.143, 95% CI: 154.9–183.7), and LDL-C levels (*p* = 0.043, η^2^ = 0.156, 95% CI: 91.5–121.9) increased significantly from T1 to T3. No significant differences were observed in the other biochemical parameters (Table 2).

Both daily salt intake (*p* = 0.017, η^2^ = 0.197, 95% CI: 6.8–10.9) and 24 h urinary sodium excretion (*p* = 0.017, η^2^ = 0.197, 95% CI: 117.7–188.9) increased significantly across tertiles. Specifically, mean salt intake rose from 8.9 ± 3.4 g/day (T1) to 15.9 ± 8.9 g/day (T3), while 24 h urinary sodium excretion increased from 96.8 ± 35.3 mmol/24 h (T1) to 151.1 ± 74.6 mmol/24 h (T3) (Table 2).

After adjustment for BMI, sex, and age, a positive and significant correlation was observed between LAP and mClCr (r = 0.40, 95% CI: 0.10–0.63, *p* < 0.05), 24 h urinary sodium excretion (r = 0.50, 95% CI: 0.23–0.70, *p* < 0.001), and daily salt intake (r = 0.50, 95% CI: 0.23–0.70, *p* < 0.001).

### 3.3. Proteinuria and Visceral Adiposity

When participants were stratified by proteinuria levels [Group 1: <150 mg/day; Group 2: ≥150 mg/day], significant metabolic and renal differences were noted (Table 3).

Subjects with proteinuria exhibited significantly higher NVAI values [1.0 (95% CI: 0.9–1.0) vs. 0.9 (95% CI: 0.8–0.9), *p <* 0.05] and elevated 24 h urinary sodium excretion (336.7 ± 194.5 vs. 187.2 ± 73.3 mmol/24 h, Cohen’s d = 1.52, 95% CI: 61.5–237.6, *p* = 0.001) as well as increased potassium excretion (82.6 ± 26.6 vs. 55.0 ± 18.8 mmol/24 h, Cohen’s d = 1.20, 95% CI: 4.4–50.8, *p* < 0.022) (Table 3).

In addition, these subjects also showed increased creatinine clearance (179.7 ± 44.9 vs. 134.8 ± 48.17 mL/min; Cohen’s d = 0.93, 95% CI: 6.7–82.0 *p* = 0.022), daily salt intake (19.6 ± 11.4 vs. 10.8 ± 4.3, Cohen’s d = 1.55, 95% CI: 3.6–13.9, *p* = 0.001), and serum ferritin concentrations (81.1 (97.2–479.6) vs. 33.0 (13.9–122.3),Cohen’s d = 1.17, *p* = 0.006) compared with those without proteinuria (Table 3).

**Figure 1:**
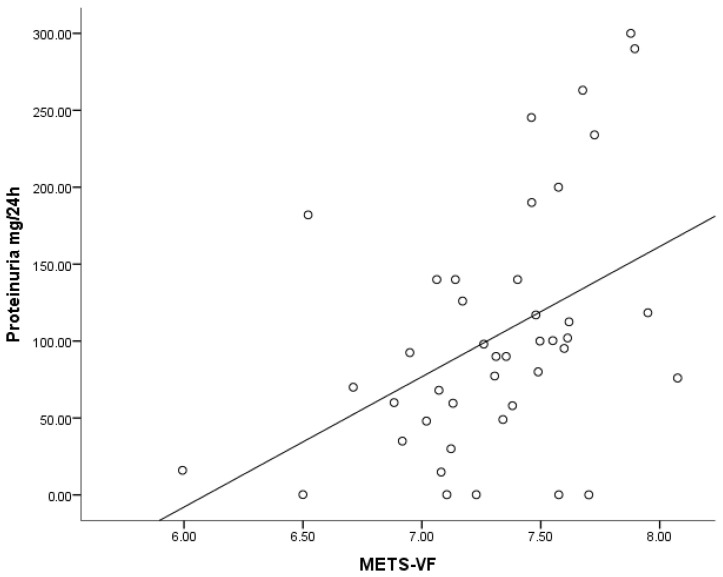
Partial linear correlation after adjustment for age, sex, and BMI between METS-VF and proteinuria mg/24 h (r = 0.50, 95% CI 0.23–0.70, *p* < 0.05).

After adjustment for BMI, sex, and age, METS-VF remained significantly correlated with 24 h urinary protein excretion (r = 0.50, 95% CI: 0.23–0.70, *p* < 0.001) in the entire group (Figure 1).

## 4. Discussion

This study explored the relationship between visceral adiposity, glomerular hyperfiltration, and proteinuria in adults with obesity. Our findings indicate that indices reflecting visceral fat accumulation—particularly LAP, NVAI, and METS-VF—are positively associated with creatinine clearance, proteinuria, and urinary sodium excretion, even after adjustment for confounders such as age and BMI. These data support the hypothesis that visceral adiposity contributes to early renal hemodynamic changes and functional hyperfiltration in obesity, potentially paving the way to CKD.

The concept that central rather than peripheral obesity plays a pivotal role in renal dysfunction is not new. Scaglione et al. [19] first demonstrated that abdominal fat distribution, more than overall obesity, was associated with alterations in renal hemodynamics. Subsequent population-based analyses confirmed that waist-to-hip ratio (WHR) and visceral fat indices correlate more strongly with hyperfiltration than BMI or waist circumference [20]. The present study aligns with these observations, emphasizing the importance of visceral fat-related metabolic activity as a pathogenic determinant of kidney injury.

Moreover, the association between mCrCl increase and higher levels of proteinuria in our population of subjects with obesity without evidence of coexisting cardiometabolic complications including diabetes mellitus, hypertension, or overt kidney disease suggests a possible mechanism of early kidney damage linked to hyperfiltration, confirming that “healthy obesity” is an independent risk factor for renal injury [21,22].

Our data also revealed that higher mClCr tertiles were associated with increased HbA1c, total cholesterol, and LDL-C levels, suggesting that metabolic dysregulation parallels renal hyperfiltration and proteinuria. Furthermore, 24 h urinary sodium excretion increased significantly, corresponding to a higher estimated daily salt intake, implicating sodium handling as an important mediator of obesity-related renal dysfunction. This is consistent with prior reports showing that sodium retention and altered pressure–natriuresis relationships in obesity contribute to hyperfiltration via activation of the renin–angiotensin–aldosterone system (RAAS) and tubuloglomerular feedback [23,24].

In addition, high salt intake inducing tubulointerstitial injury [25] could be involved in hypertension-independent kidney damage.

Another finding that emerged from our study was the significantly higher urinary potassium excretion observed in participants with proteinuria. Although the causes of this finding cannot be directly inferred from our data, several speculative hypotheses can be proposed.

First, the increase in urinary potassium excretion could reflect differences in dietary quality between the two groups, even though their caloric intake was the same, as participants with proteinuria also had markedly higher urinary sodium excretion and estimated salt intake, leading to the hypothesis of a likely higher consumption of ultra-processed foods (UPFs) in this population. In this regard, it must also be considered that visceral obesity, which is significantly more prevalent in subjects with proteinuria, has been found to be associated with a higher consumption of UPFs [11], characterized by a high sodium, phosphorus, and potassium content.

In fact, urinary potassium excretion is commonly considered a surrogate marker of dietary potassium intake [26].

Second, obesity-related hyperfiltration may in itself contribute to increased renal potassium secretion. Increased glomerular filtration and distal sodium delivery can stimulate potassium secretion in the distal nephron through flow-dependent mechanisms, thereby increasing urinary potassium losses [27]. In this context, the higher creatinine clearance observed in individuals with proteinuria may reflect hemodynamic changes that affect not only sodium delivery but also potassium secretion.

The main characteristic of this phenomenon is the one mentioned above, the RAAS, which may represent an additional key factor in kidney damage through the production of angiotensinogen derived from visceral fat cells and through the stimulation of the sympathetic nervous system [28,29,30]. Increased aldosterone activity enhances sodium reabsorption in the distal tubules while promoting potassium secretion, which may contribute to the concomitant increase in urinary sodium and potassium excretion observed in our cohort [31].

Although these hypotheses require confirmation in prospective studies that include direct assessment of dietary potassium intake and RAAS activity, our results suggest that abnormalities in potassium homeostasis may accompany the early renal and tubular changes associated with obesity-related hyperfiltration and proteinuria.

The observed correlations between visceral adiposity indices and both proteinuria and sodium excretion further suggest a common mechanistic link. Visceral adipose tissue is an active endocrine organ that releases inflammatory cytokines (e.g., IL-6, TNF-α) and adipokines (e.g., leptin, resistin) capable of altering renal hemodynamics and promoting endothelial dysfunction [32,33]. Chronic low-grade inflammation, insulin resistance, and lipotoxicity contribute to podocyte injury, mesangial expansion, and glomerulosclerosis [34,35]. These effects may be exacerbated by high dietary sodium intake, which amplifies glomerular hypertension and intrarenal RAAS activation and increased urinary protein excretion.

In our cohort, individuals with proteinuria exhibited higher levels of NVAI, METS-VF, and LAP—indices integrating both anthropometric and biochemical markers of metabolic dysfunction. These findings support the use of visceral adiposity indices as practical tools for assessing obesity-related kidney injury risk. Similar associations between these indices and CKD have been reported in different populations [13,36,37].

An important secondary observation was the significantly elevated serum ferritin levels in participants with proteinuria ≥ 150 mg/day compared to those without proteinuria. Ferritin is commonly interpreted as a marker of systemic inflammation, potentially reflecting the increased production of pro-inflammatory cytokines (IL-6, TNF-α) released by visceral adipose tissue. This finding warrants cautious interpretation owing to ferritin’s multifactorial determinants. Elevated ferritin in our cohort may reflect one or more of the following: (1) systemic inflammation and oxidative stress secondary to visceral adiposity and renal hemodynamic perturbations; (2) metabolic dysfunction and insulin resistance, which are independently associated with ferritin elevation; (3) hepatic steatosis (non-alcoholic fatty liver disease, NAFLD), which may be associated with visceral obesity and may impair hepcidin-mediated iron regulation through mechanisms involving inflammation and insulin resistance; or (4) altered iron metabolism secondary to chronic low-grade inflammation or renal dysfunction itself [25,34].

The specificity and reliability of ferritin as an isolated inflammatory biomarker are limited: ferritin is an acute-phase reactant but lacks biological specificity, being influenced by iron stores, acute infections, liver disease, and hemolysis. Furthermore, serum ferritin exhibits considerable intraindividual variability, rendering it suboptimal as a single marker of chronic inflammation. A more robust assessment of the inflammatory phenotype in our population would require complementary high-sensitivity markers such as high-sensitivity C-reactive protein (hsCRP) or the direct measurement of circulating pro-inflammatory cytokines (IL-6, TNF-α) [32].

Nonetheless, the concomitant elevation of ferritin, proteinuria, creatinine clearance, and visceral adiposity indices in our cohort is consistent with a state of metabolic and hemodynamic dysregulation in which ferritin may serve as one component of a broader inflammatory–metabolic milieu. Future prospective studies integrating ferritin with comprehensive inflammatory and metabolic biomarker panels would help elucidate the relative contribution of these mechanistic pathways to obesity-related glomerular hyperfiltration and early chronic kidney disease.

Several pathophysiological mechanisms have been proposed to explain how visceral adiposity promotes hyperfiltration and subsequent CKD. These include hemodynamic alterations, such as afferent arteriolar dilation and increased renal plasma flow; neurohumoral activation, including sympathetic overactivity and upregulation of the RAAS; metabolic toxicity, resulting from ectopic lipid accumulation in renal tissue (“lipotoxicity”); and inflammatory and oxidative stress pathways, leading to podocyte dysfunction and interstitial fibrosis. Together, these mechanisms contribute to the transition from adaptive hyperfiltration to irreversible glomerular injury, characterized histologically by glomerulomegaly and segmental sclerosis [38].

The relationship between sodium balance and glomerular hemodynamics deserves particular attention. Experimental studies show that acute salt loading can increase GFR independently of systemic RAAS activation [39]. However, chronic excessive sodium intake, typical of high-calorie Western diets, may promote persistent intraglomerular hypertension and accelerate kidney damage, particularly in individuals with visceral obesity. This interplay between sodium and adiposity highlights the multifactorial nature of obesity-related glomerulopathy.

The strengths of this study include the comprehensive assessment of multiple validated adiposity indices and the evaluation of both renal and metabolic parameters within a well-characterized cohort.

However, limitations should be acknowledged.

In addition to the hypothesis of an association between a high intake of UPFs and increased urinary excretion of potassium and sodium, it would have been desirable to assess UPF daily intake using specifically designed questionnaires. Moreover, GFR estimation was based on creatinine clearance, which may overestimate true filtration in obesity due to increased creatinine generation. Cystatin-C-based estimates could have provided greater precision. Additionally, the cross-sectional design precludes causal inference.

The findings suggest that monitoring visceral adiposity and dietary sodium intake could improve the early identification of obese individuals at risk of kidney damage. Lifestyle interventions targeting visceral fat reduction and sodium restriction may represent effective strategies to mitigate renal hyperfiltration and delay CKD onset.

Future longitudinal studies should evaluate whether reductions in visceral adiposity indices translate into improved renal outcomes, particularly in patients with early proteinuria or subclinical hyperfiltration.

In summary, in this study, we demonstrated a clear association between visceral adiposity indices and markers of renal hyperfiltration, proteinuria, and sodium excretion in adults with obesity. These findings suggest that visceral fat accumulation, rather than general obesity, plays a pivotal role in early renal hemodynamic changes and metabolic stress, fostering kidney injury.

## 5. Conclusions

Our results support the potential clinical utility of visceral adiposity indices—such as LAP, NVAI, and METS-VF—as non-invasive markers for identifying individuals at risk of obesity-related glomerular hyperfiltration and early CKD and suggest that monitoring visceral adiposity and dietary sodium intake could improve early identification of obese individuals at risk of kidney damage.

Given the concomitant role of sodium handling abnormalities, lifestyle interventions targeting visceral fat reduction and dietary sodium restriction should be encouraged in clinical practice.

Further longitudinal studies are warranted to elucidate the causal pathways linking visceral adiposity and renal dysfunction and to verify whether an improvement in these adiposity indices translates into improved renal outcomes, particularly in subjects with early proteinuria or subclinical hyperfiltration.

## Figures and Tables

**Table 1:** Baseline anthropometric body composition and clinical characteristics of the study population.

Study Population Features	
**n°**	43
**Male, %**	37.2%
Age, years *	37.0 ± 10.7
Weight, Kg *	131.0 ± 29.1
BMI, Kg/m^2^ *	46.3 ± 8.1
WC, cm *	121.4 ± 15.8
Energy intake, Kcal/day *	3589.9 ± 1084.3
LAP *	87.9 ± 48.5
NVAI **	0.9 (0.8–0.9)
VAI *	2.4 ± 2.8
TyG *	8.3 ± 1.2
BRI *	8.7 ± 2.5
METS-VF *	7.3 ± 0.4
FM, % *	46.6 ± 7.7
FFM, % *	53.4 ± 7.7
TBW % *	40.0 ± 6.0
Phase angle, Φ *	6.3 ± 0.9
Proteinuria, mg/24 h *	129.9 ± 221.4
Natriuresis, mEq/24 h *	209.6 ± 110.0
Creatinine clearance, mL/min *	143.0 ± 50.1

Continuous variables are expressed as * mean ± SD or ** median and interquartile range (IQR). Categorical variables are expressed as numbers and percentages. Abbreviations are as follows: BMI, body mass index; WC, waist circumference; FFM, Fat-Free Mass; FM, Fat Mass; LAP, Lipid Accumulation Product; VAI, Visceral Adiposity Index; NVAI, New Visceral Adiposity Index; TyG, Triglyceride–Glucose Index; BRI, Body Roundness Index; METS-VF, Metabolism Score for Visceral Fat.

**Table 2:** Anthropometric, body composition, clinical, biochemical, and visceral adiposity parameters according to creatinine clearance tertiles.

	T1 (<121.4) n. 14	T2 (≥121.4 and <159.6) n. 15	T3 (≥159.6) n. 14	*p*-Value
**Male, %**	21.4	33.3	857.1	0.137
**Age, years**	32.0 ± 8.6	37.4 ± 12.3	41.5 ± 9.2	0.053
**Kcal/day**	3900.3 ± 1181.2	3184.1 ± 621.9	3714.2 ± 1294.9	0.182
**Intake CHO, % of tot food**	43.4 ± 6.9	43.7 ± 8.8	44 ± 9.5	0.969
**Intake PRO, % of tot food**	16.9 ± 2.0	17.2 ± 3.8	16.9 ± 3.1	0.962
**Intake LIP, % of tot food**	39.5 ± 6.1	39.3 ± 6.8	38.8 ± 8.3	0.959
**Weight, Kg**	120.9 ± 24.1	127.2 ± 27.0	145.3 ± 29.2	0.055
**BMI, Kg/m^2^**	44.5 ± 8.3	44.5 ± 6.4	50.1 ± 8.9	0.111
**WC, cm**	114.4 ± 18.1	121.2 ± 14.9	128.7 ± 11.7	0.056
**FM, %**	47.7% ± 5.9	46.1% ± 7.4	45.9 ± 9.8	0.805
**FFM, %**	52.2% ± 5.9	55.0% ± 7.4	55.2 ± 9.8	0.805
**TBW %**	38.7% ± 5.9	40.5% ± 7.4	40.7 ± 9.8	0.625
**Phase angle, Φ**	6.2 ± 0.7	6.3 ± 0.9	6.3 ± 1.1	0.931
**LAP**	61.9 ± 35.3	92.4 ± 38.8	109.3 ± 58.6 °	*0.028*
**NVAI**	0.8 (0.5–0.9)	0.9 (0.8–0.9)	1.0 (0.9–1.0) °	*0.006*
**VAI**	1.5 ± 0.9	3.6 ± 4.4	2.1 ± 1.7	0.142
**TyG**	8.3 ± 0.4	8.3 ± 1.4	8.3 ± 1.5	0.988
**BRI**	8.0 ± 3.2	8.5 ± 2.2	9.5 ± 1.8	0.271
**METS-VF**	7.0 ± 0.4	7.3 ± 0.2	7.6 ± 0.3 °	*0.007*
**Proteinuria, mg/24 h**	61.0 ± 48.2	110.7 ± 61.7	135.8 ± 102.1 °	*0.033*
**Natriuresis, mEq/24 h**	96.8 ± 35.3	139.5 ± 58.9	151.1 ± 74.6 °	*0.017*
**Salt intake, g/day**	8.9 ± 3.4	11.8 ± 4.3	15.9 ± 8.9 °	*0.017*
**Urea, mg/dL**	29.3 ± 8.9	31.2 ± 6.1	31.3 ± 8.3	0.750
**Creatinine, mg/dL**	0.8 ± 0.1	0.8 ± 0.2	0.8 ± 0.2	0.910
**Uric acid, mg/dL**	5.7 (3.7–6.3)	5.8 (5.0–6.4)	5.6 (4.8–7.1)	0.557
**Basal insulin, mU/mL**	27.8 ± 4.8	30.8 ± 5.8	32.3 ± 7.4	0.872
**Glucose, mg/dL**	90.1 ± 15.3	91.7 ± 11.3	92.7 ± 10.6	0.861
**Homa index**	6.4 ± 5.3	7.2 ± 5.5	7.5 ± 6.9	0.883
**HbA1c, %**	5.1 ± 0.4	5.5 ± 0.4 *	5.6 ± 0.2 °	*0.004*
**Tot-C, mg/dL**	169.3 ± 24.9	183.4 ± 25.9	198.7 ± 38.9 °	*0.049*
**TG, mg/dL**	97.7 ± 42.6	137.1 ± 58.4	144.9 ± 77.8	0.105
**LDL-C, mg/dL**	106.7 ± 26.3	114.6 ± 22.6	135.1 ± 34.3 °	*0.043*
**HDL-C, mg/dL**	50.1 ± 9.6	47.3 ± 11.5	48.3 ± 9.2	0.748
**Ferritin, ng/mL**	28.8 (12.1–74.9)	48.3 (19.4–90)	130.7 (19.3–214.2)	0.295
**SBP, mmHg**	125.1 ± 12.7	130.7 ± 12.4	135.7 ± 10.2	0.072
**DBP, mmHg**	80.3 ± 8.4	85.0 ± 11.3	85.3 ± 6.9	0.280

Data are expressed as mean ± SD or median and interquartile range (IQR). ° *p* < 0.05 T3 vs. T1, * *p* < 0.05 T2 vs. T1 after Bonferroni correction. Categorical variables are expressed as numbers and percentages. Italics are used to highlight statistical significance. Abbreviations are as follows: Tot-C, Tot Cholesterol; LDL-C, Low-Density Lipoprotein–Cholesterol; HDL-C, High-Density Lipoprotein–Cholesterol; TG, Triglyceride; HbA1c, glycated hemoglobin; SBP, Systolic Blood Pressure; DBP, Diastolic Blood Pressure; BMI, body mass index; WC, waist circumference; FFM, Fat-Free Mass; FM, Fat Mass; LAP, Lipid Accumulation Product; VAI, Visceral Adiposity Index; NVAI, New Visceral Adiposity Index; TyG, Triglyceride–Glucose Index; BRI, Body Roundness Index; METS-VF, Metabolism Score for Visceral Fat; CHO, carbohydrate; PRO, protein; LIP, lipid.

**Table 3:** Anthropometric, body composition, clinical, biochemical, and visceral adiposity parameters among proteinuria groups.

	G 1 (<150.0) n. 35	G 2 (≥150.0) n. 8	*p*-Value
**Male, %**	29.4	66.7	0.037
Age, years *	36.7 ± 10.8	39.1 ± 11.1	0.544
Kcal/day *	3610.9 ± 1139.2	3497.8 ± 859.9	0.794
**Intake CHO, % of tot food**	43.8 ± 8.7	43.5 ± 6.4	0.926
**Intake PRO, % of tot food**	16.8 ± 2.9	18.0 ± 3.2	0.309
**Intake LIP, % of tot food**	39.4 ± 6.8	38.3 ± 8.0	0.676
Weight, Kg *	128.1 ± 27.9	143.8 ± 26.9	0.156
BMI, Kg/m^2^ *	46.1 ± 8.2	47.4 ± 8.2	0.690
WC, cm *	119.9 ± 15.9	127.7 ± 15.1	0.215
FM, % *	47.6 ± 7.3	42.3 ± 8.3	0.079
FFM, % *	52.4 ± 7.3	57.7 ± 8.3	0.079
TBW % *	39.1 ± 5.5	43.9 ± 6.7	0.038
Phase angle, Φ *	6.2 ± 0.9	6.4 ± 0.7	0.680
LAP *	84.2 ± 40.3	103.4 ± 76.2	0.320
NVAI **	0.9 (0.8–0.9)	1.0 (0.9–1.0)	0.044
VAI *	2.4 ± 2.9	2.3 ± 2.2	0.912
TyG *	8.4 ± 0.9	8.0 ± 1.9	0.444
BRI *	8.6 ± 2.5	8.9 ± 2.4	0.707
METS-VF *	7.2 ± 0.4	7.5 ± 0.4	0.095
Natriuresis, mEq/24 h *	187.2 ± 73.3	336.7 ± 194.5	0.001
Salt intake, g/day *		19.6 ± 11.4	0.001
mClCr, mL/min **	134.8 ± 48.1	179.7 ± 44.9	0.022
Kaliuresis, mEq/24 h *	55.0 ± 18.8	82.6 ± 26.6	0.022
Urea, mg/dL *	30.4 ± 8.2	31.4 ± 5.5	0.755
Creatinine, mg/dL *	0.8 ± 0.2	0.8 ± 0.1	0.514
Uric acid, mg/dL **	5.7 (4.7–6.7)	5.6 (4.6–6.1)	0.507
Basal insulin, mU/mL *	30.1 ± 21.0	31.3 ± 29.3	0.892
Glucose, mg/dL *	91.6 ± 12.8	91.0 ± 10.4	0.893
Homa Index *	6.9 ± 5.4	7.3 ± 7.9	0.879
HbA1c, % *	5.3 ± 0.4	5.6 ± 0.3	0.077
Tot-C, mg/dL *	182.3 ± 33.4	190.1 ± 26.4	0.542
TG, mg/dL *	123.9 ± 58.3	137.8 ± 85.4	0.582
LDL-C, mg/dL *	115.4 ± 30.4	127.3 ± 21.0	0.333
HDL-C, mg/dL *	49.8 ± 9.7	43.2 ± 10.0	0.097
Ferritin, ng/mL **	33.0 (13.9–122.3)	81.1 (97.2–479.6)	0.036
SBP, mmHg *	129.5 ± 12.1	135 ± 13.1	0.257
DBP, mmHg *	83.4 ± 9.4	84.4 ± 9.0	0.797

Data are expressed as * mean ± SD or ** median and interquartile range (IQR). Categorical variables are expressed as numbers and percentages. Abbreviations are as follows: Tot-C, Tot Cholesterol; LDL-C, Low-Density Lipoprotein–Cholesterol; HDL-C, High-Density Lipoprotein–Cholesterol; TG, Triglyceride; HbA1c, glycated hemoglobin; SBP, Systolic Blood Pressure; DBP, Diastolic Blood Pressure; BMI, body mass index; WC, waist circumference; FFM, Fat-Free Mass; FM, Fat Mass; LAP, Lipid Accumulation Product; VAI, Visceral Adiposity Index; NVAI, New Visceral Adiposity Index; TyG, Triglyceride–Glucose Index; BRI, Body Roundness Index; METS-VF, Metabolism Score for Visceral Fat; CHO, carbohydrate; PRO, protein; LIP, lipid.

## Data Availability

The data are stored in a database at the Department of Clinical Medicine and Surgery, Nutrition Physiology Unit, University Federico II of Naples, Naples 80131, Italy. It is available upon request to Bruna Guida due to privacy restrictions, who is a co-author of the paper.

## Supplementary Materials

The following supporting information can be downloaded at https://www.mdpi.com/article/10.3390/nu18142233/s1. Table S1: Formulas and interpretation of adiposity indices used in the study.

## Abbreviations

The following abbreviations are used in this manuscript:

CKD	Chronic kidney disease
mClCr	measured creatinine clearance
BMI	Body mass index
WC	Waist circumference
BIA	Bioelectrical impedance analysis
FM	Fat Mass
FFM	Fat-Free Mass
PA	Phase angle
WHtR	Waist-to-height ratio
WHR	Waist-to-hip-ratio
HbA1c	Glycated hemoglobin
Tot-C	Total cholesterol
LDL-C	Low-Density Lipoprotein–Cholesterol
HDL-C	High-Density Lipoprotein–Cholesterol
TG	Triglyceride
SBP	Systolic Blood Pressure
DBP	Diastolic Blood Pressure
VAI	Visceral Adiposity Index
NVAI	New Visceral Adiposity Index
LAP	Lipid Accumulation Product
TyG	Triglyceride–Glucose Index
BRI	Body Roundness Index
METS-VF	Metabolic Score for Visceral Fat
RAAS	Renin–Angiotensin–Aldosterone System
