# Supplementary material for: Hyperfiltration in Obesity: The Vicious Link Between Visceral Fat, Proteinuria, and Sodium Excretion
Source: Nutrients. 2026 Jul 9;18(14):2233. doi: 10.3390/nu18142233 (PMC13416162; doi:10.3390/nu18142233)
Supplement: Supplementary file 1 [file nutrients-18-02233-s001.zip › nutrients-4372909-supplementary.pdf]

Supplementary Table S1. Formulas and interpretation of adiposity indices used in the study

Index name	Formula	Reference	Clinical interpretation
**Waist-to-Height Ratio (WHtR)**	WC / Height (both in cm)	Di Lorenzo et al., 2024	Simple anthropometric indicator of central obesity; values >0.5 are associated with increased cardiometabolic and renal risk.
**Visceral Adiposity Index (VAI)**	Men: (WC / [39.68 + (1.88 × BMI)]) × (TG / 1.03) × (1.31 / HDL-C)<br>Women: (WC / [36.58 + (1.89 × BMI)]) × (TG / 0.81) × (1.52 / HDL-C)	Amato et al., 2010	Reflects visceral fat function and insulin resistance; higher values indicate metabolic and renal risk.
**New Visceral Adiposity Index (NVAI)**	Men: 1 / [1 + e^−(−21.858 + 0.099×Age + 0.10×WC + 0.12×MAP + 0.006×TG − 0.077×HDL-C)]<br>Women: 1 / [1 + e^−(−18.765 + 0.058×Age + 0.14×WC + 0.057×MAP + 0.004×TG − 0.057×HDL-C)]	Jin et al., 2023	Incorporates hemodynamic and lipid parameters to better estimate visceral fat burden; higher NVAI correlates with hyperfiltration and CKD risk.
**Lipid Accumulation Product (LAP)**	Men: (WC − 65) × TG<br>Women: (WC − 58) × TG	Kahn, 2005	Surrogate marker of ectopic fat accumulation; strongly associated with insulin resistance and renal dysfunction.
**Triglyceride–Glucose Index (TyG)**	ln [TG (mg/dL) × Glucose (mg/dL) / 2]	Kurniawan et al., 2024	Marker of insulin resistance and metabolic risk; elevated values predict CKD and cardiovascular disease.

**Body Roundness Index (BRI)**	$364.2 - 365.5 \times \sqrt{(1 - (WC / (2\pi))^2 / (0.5 \times Height)^2)}$	Zhou et al., 2025	Estimates body shape and fat distribution; higher BRI indicates greater central obesity.
**Metabolic Score for Visceral Fat (METS-VF)**	$4.466 + 0.011 \times (\ln(METS\text{-}IR))^3 + 3.329 \times (\ln(WHtR))^3 + 0.319 \times Sex + 0.594 \times \ln(Age)$	Zhou et al., 2025	Comprehensive indicator of metabolic–visceral fat dysfunction; correlated with cardiometabolic–renal (CKM) syndrome and CKD.
